# The Effects of K-BNNS Nanoparticles on PD Characteristics of Composite Aramid Paper

**DOI:** 10.3390/nano16040249

**Published:** 2026-02-14

**Authors:** Yan-Hong Chen, Xiao-Nan Li, Wen-Xu Zhang, Tong Qin, Qi-Kun Cheng, Bin Wu

**Affiliations:** 1 School of Electrical Engineering, Northwest Minzu University, Lanzhou 730030, China; 15193195460@163.com; 2 School of Automation and Electrical Engineering, Lanzhou University of Technology, Lanzhou 730050, China; 18993993425@163.com; 3 Urumqi Power Supply Company, Grid Xinjiang Electric Power Company, Urumqi 830001, China; 222085801038@lut.edu.cn; 4 Electric Power Dispatching Center State, Grid Gansu Electric Power Company, Lanzhou 730030, China; 13893624523@163.com

**Keywords:** partial discharge, surface charge, charge transport, K-BNNS, composite aramid paper

## Abstract

Aramid paper serves as an insulating material in high-frequency power electronic transformers, and the incorporation of composite K-BNNS particles has been shown to enhance the insulating properties of aramid paper. Partial discharge (PD) is a predominant phenomenon that can lead to insulation failure in high-frequency transformers. Therefore, this paper primarily investigates the PD performances of different nanoparticle doping concentrations on aramid paper. Firstly, composite aramid papers containing K-BNNSs at different concentrations are prepared, namely 5%, 8%, 10% and 13%, respectively. Then, the experimental platforms of PD for composite aramid paper are established, and the PD performances, surface potentials, and hydrogen bonds under different high-frequency applied voltages are discussed. The experiment results show that the composite aramid paper with 10% K-BNNSs nanoparticle content has the optimal insulation performance. In the needle-plate and column-plate models, the PD amplitude decreases by 65.35% and 27.33%, respectively, when compared with non-doped aramid paper. Moreover, the breakdown voltage improves by 32.2% and 38.5%, respectively. After that, the influence mechanisms of residual charges and hydrogen bonds on the PD characteristics of composite aramid paper are analyzed. The results obtained in this paper can provide important reference for the design and selection of insulation materials for high-frequency transformers.

## 1. Introduction

Power electronic transformers consist of power electronic converters and high-frequency transformers, which offer advantages such as a compact size, lightweight design, and high power density. In recent years, these devices have attracted considerable attention in the field of power electronics. High-frequency pulse voltages generated by power electronic converters often impinge upon the insulation characteristics of high-frequency transformers, thereby causing premature insulation failure. Existing studies have shown that high-frequency PD represents the primary cause of insulation failure in high-frequency transformers [1]. Currently, insulation in high-frequency transformers is primarily achieved through dry-type or oil-immersed methods. Oil-immersed configurations, with their superior heat dissipation and insulation properties, facilitate the development of high-frequency transformers with higher power, voltage, and capacity ratings. Given that aramid paper serves as the inter-turn insulation material in oil-immersed high-frequency transformers, the optimization of its PD characteristics under high-frequency operating conditions is of considerable research significance.

Research conducted by some scholars has primarily focused on high-frequency discharge characteristics, charge transport properties, and material optimization. With regard to high-frequency PD characteristics and the associated mechanisms, Han et al. [2] investigated the effects of different pulse waveforms on the surface discharge characteristics and insulation lifetime of polyimide films. Their findings indicated that the partial discharge initiation voltage varies little under different voltage waveforms, whereas the phase distribution and statistical characteristics of partial discharges exhibit significant changes. Furthermore, the aging rate under bipolar waveforms is higher than that under unipolar waveforms. Zhang et al. [3] employed finite element and molecular simulation methods to investigate the insulation failure mechanisms of polyimide under high-frequency electrical stress. Zhao, Li, and Zhang et al. [4,5,6,7,8] investigated the breakdown characteristics and PD properties of epoxy resin under high-frequency square-wave voltages. It was found that the flashover breakdown voltage, partial discharge initiation voltage, partial discharge amplitude, and insulation lifetime decrease significantly with increasing frequency, whereas the breakdown time is shortened. Moreover, the high-frequency thermal effect has been identified as the primary cause of the reduced breakdown voltage and shortened insulation lifetime. In contrast, Jiang et al. [9] observed that partial discharge amplitude and discharge repetition rate increase with frequency. Li et al. [10] investigated the PD characteristics of oil-paper insulation under high-frequency electrical stress and identified a “frequency-induced inflection point” effect in the discharge behavior of high-frequency oil-paper insulation.

Regarding charge transport characteristics, Li et al. [11] investigated the surface charge transport properties of oil-paper insulation under high-frequency pulse voltages. Their study revealed that the initial surface charge and trap density of high-frequency oil-paper specimens decrease with increasing frequency, whereas the surface potential decay rate initially increases and then decreases. Ren et al. [12] investigated the effect of unipolar square-wave voltage on the space charge characteristics of polyimide. Their findings indicated that internal charge accumulation in polyimide materials under square-wave voltage is greater than that under DC voltage, and that even higher charge accumulation and more severe electric field distortion occur under negative square-wave voltage.

Regarding material optimization, Han et al. [13] developed an electro-thermal coupled discharge breakdown phase-field model to study the evolution of breakdown pathways in polyimide subjected to high-frequency electrical stress. Their study revealed that, as frequency and temperature increase, the development of discharge breakdown pathways accelerates, with Joule heating gradually becoming the dominant factor. Asif et al. [14] investigated the influence of nanoparticles on the aging mechanism of polyimide (PI) and found that PI/TiO_2_ nanocomposites exhibit fewer defects, thereby suppressing the occurrence of PD. Xing et al. [15] developed disiloxane-modified polyimide films and observed that a 5% content significantly enhances the high-frequency surface dielectric strength of PI. Li et al. [16] prepared epoxy resin composites modified with dopamine-grafted nanoscale boron nitride (h-BN). Their findings showed that doping at a mass fraction of 10% yields the highest charge dissipation rate, with the high-frequency surface flashover voltage increasing by 14.73% compared to pure epoxy resin. Song et al. [17] prepared epoxy composites using a composite filler consisting of small SiO_2_ particles and large BN particles, thereby significantly enhancing both thermal conductivity and high-frequency electrical insulation properties.

Building on these studies, Li et al. [18] prepared composite aramid paper and found that its optimal PD resistance is achieved with a 10% content of the composite particle K-BNNSs. However, the mechanism by which K-BNNS nanoparticles influence the partial discharge characteristics of composite aramid paper remains unclear. Furthermore, existing research [19] suggests that composite particles alter the charge transport properties of the composite material, thereby influencing its partial discharge characteristics. Nevertheless, this hypothesis remains largely unverified by experimental evidence and theoretical studies. To address this gap, the present study established an experimental platform for high-frequency partial discharge and charge transport in composite aramid paper, as well as a terahertz detection platform. The low-frequency vibrations of terahertz waves can precisely reflect intermolecular forces such as hydrogen bonding, and terahertz spectroscopy can effectively evaluate the uniformity, dielectric properties, and molecular structural changes in insulating materials, providing key support for materials-performance research. Using the step-up voltage method and the constant voltage method, the high-frequency partial discharge characteristics and charge transport properties of composite aramid paper have been investigated. In addition, the mechanism by which K-BNNS nanoparticles influence the partial discharge characteristics of the composite aramid paper has been explored.

## 2. Experimental Platform

### 2.1. PD Experiment

**Figure 1:**
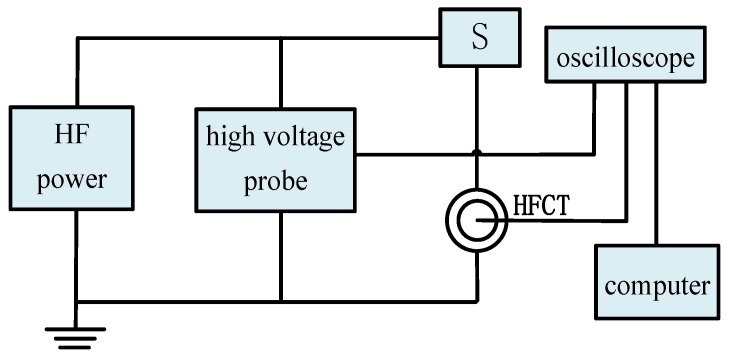
High-frequency PD test platform.

**Figure 2:**
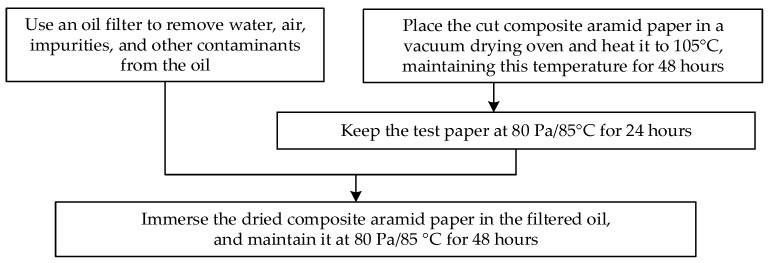
Specimen processing flowchart.

A high-frequency PD test platform for composite aramid paper was established to investigate the influence of K-BNNS nanoparticles on the partial discharge characteristics of composite aramid paper. The test platform is illustrated in Figure 1. It comprises a high-frequency power supply, a high-voltage probe, the test model (S), a high-frequency current transformer (HFCT), and an oscilloscope. The parameters of the relevant equipment are detailed in Table 1. Prior to partial discharge testing, background noise measurements and specimen pretreatment were performed. Herein, Karamay No.25 mineral oil was adopted as the filtered oil, and the mass fraction of KH-550 was set to 4%. The specimen pretreatment process is shown in Figure 2. The composite aramid papers illustrated in Figure 2 were selected from composite insulating papers prepared according to Reference [18], with filler contents of 0 wt%, 5 wt%, 8 wt%, 10 wt%, and 13 wt%. These specimens are designated as F-0, F-5, F-8, F-10, and F-13, respectively.

**Figure 3:**
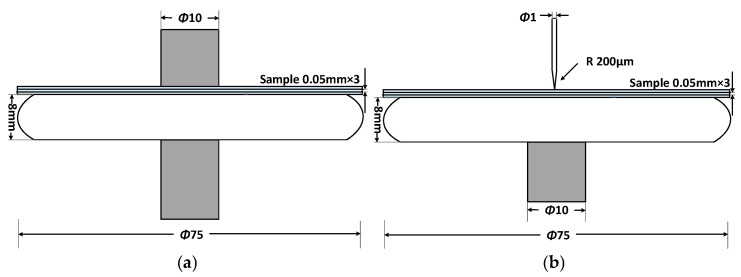
Typical defect models in transformers: (**a**) Column-plate model; (**b**) Pin-plate model.

**Figure 4:**
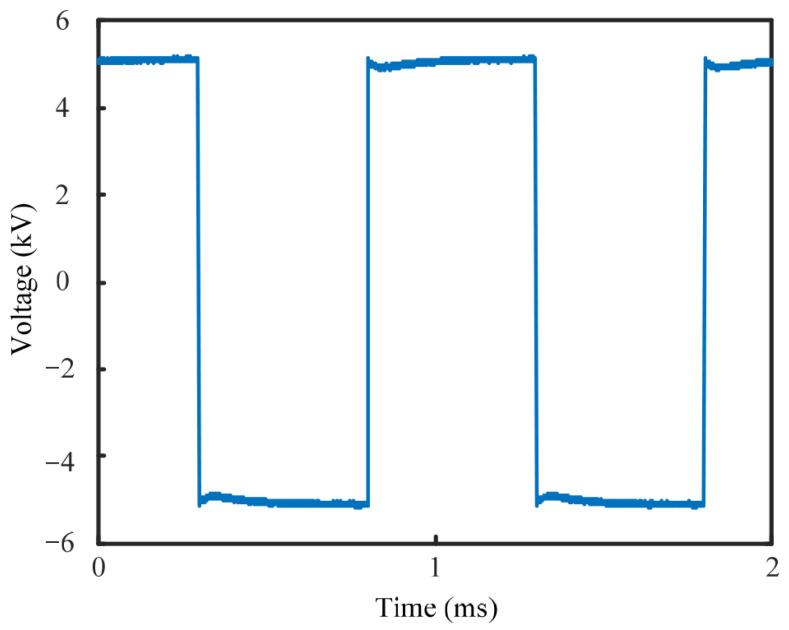
High-frequency test voltage.

Based on the actual operating conditions of high-frequency transformers, existing research, and relevant standards [20,21], needle-plate and column-plate defect models were designed, as shown in Figure 3. The output voltage waveform of the high-frequency power supply is shown in Figure 4. Subsequently, high-frequency partial discharge experiments were conducted using the step-up voltage method and the constant voltage method, with the applied test conditions detailed in Table 2.

### 2.2. Other Experiments

#### 2.2.1. Testing of Surface Charge

**Figure 5:**
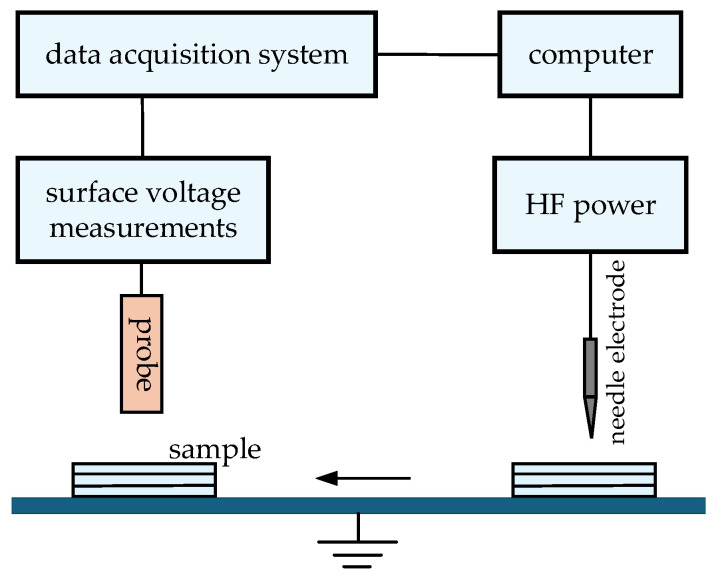
Surface potential test system under a high-frequency square wave.

To investigate the effects of different nanoparticle concentrations on the charge transport properties and PD characteristics of composite aramid paper, a surface potential measurement platform was constructed for composite aramid paper under high-frequency pulse voltages, as shown in Figure 5. The platform comprises several components, including a high-frequency power supply, a needle electrode, the sample, a probe, a surface potential meter, a data acquisition card, and a computer. The technical specifications of the equipment and the experimental conditions are presented in Table 3 and Table 4, respectively. The high-frequency power supply was used to apply voltage to the specimen via the needle electrode for corona charging. The distance between the needle electrode and the specimen was maintained at 8 mm. After a 10 min charging period, the voltage was discontinued, and the specimen was rapidly transferred to a position 3 mm below the fixed probe for measurement of its surface potential distribution. The surface potential meter, connected to the probe and the data acquisition card, recorded the real-time variation in the surface potential.

Subsequently, the surface potential value of oil-impregnated composite aramid paper by means of the active electrostatic probe method previously delineated is measured. And the charge density *σ* at the point to be measured can be obtained by inverse calculation using the linear scaling method [22], as follows:

σ = *με*_0_*ε_r_*/*d* (1)

where *μ* is the potential of the point to be measured, *ε*_0_ is the vacuum dielectric constant; *ε_r_* is the relative dielectric constant of the material; *d* is the thickness of the material.

From Equation (1), it can be observed that the charge density of the point under consideration is positively and linearly correlated with the surface potential value of said point. Furthermore, it is evident that the potential distribution can be a reflection of the charge distribution.

#### 2.2.2. Testing of Hydrogen Bonding

**Figure 6:**
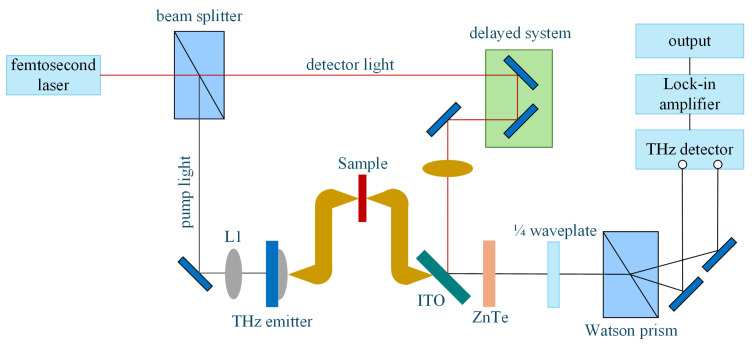
Transmitted terahertz time-domain spectroscopy system.

Previous studies [10,18] have speculated that the addition of a silane coupling agent KH-550 to the composite particles could facilitate the formation of hydrogen bonds between the composite particles and the aramid matrix; however, there has been a lack of relevant experimental verification. To verify this hypothesis, a terahertz detection platform for composite aramid paper was constructed, leveraging the ability of terahertz waves to probe deeply into the structure and strength variations of hydrogen bonds within the material [23,24], as shown in Figure 6 [25,26]. The platform primarily comprises a femtosecond laser, a terahertz radiation source, a terahertz radiation detection device, and a time delay control system. During operation, the pump light and probe light generated by the femtosecond laser are modulated and directed along their respective paths to excite terahertz radiation, ensuring synchronous arrival at the detection device. By precisely controlling the time delays between the probe light and terahertz pulses, terahertz waveforms can be effectively captured and recorded, enabling an in-depth study of the sample properties.

## 3. PD Characteristics of Composite Aramid Paper

### 3.1. The Amplitude of PD

**Figure 7:**
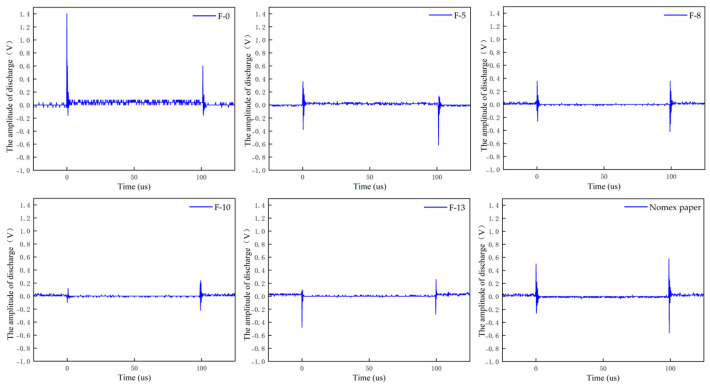
PD amplitude of needle-plate model.

**Figure 8:**
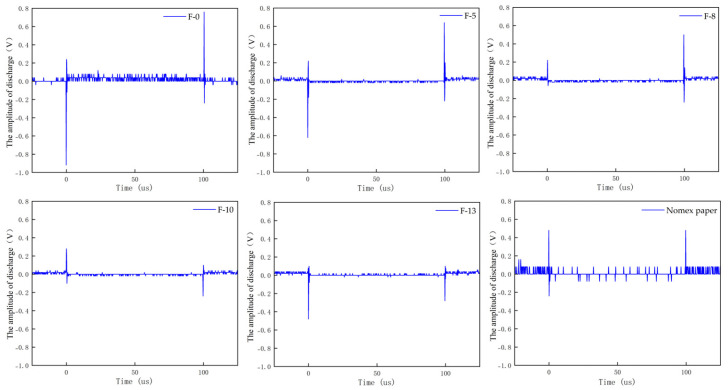
PD amplitude of column-plate model.

**Figure 9:**
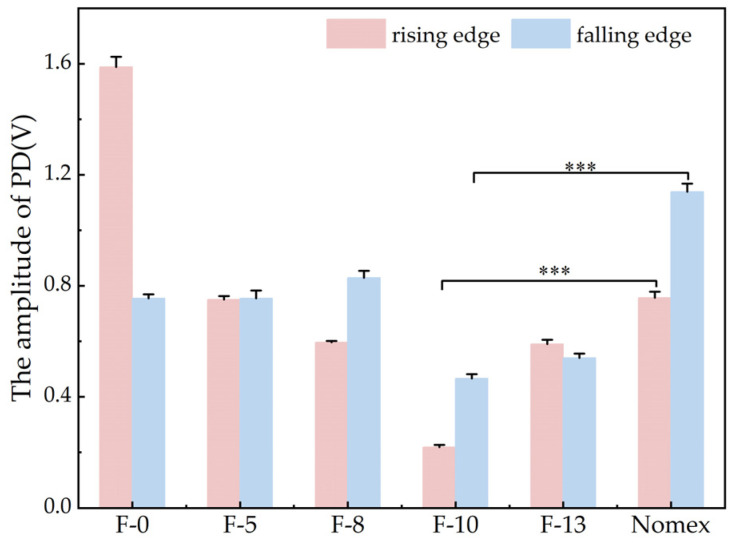
Variation curves of PD amplitude at the rising and falling edges for the needle-plate model (*** refers to p<0.001).

**Figure 10:**
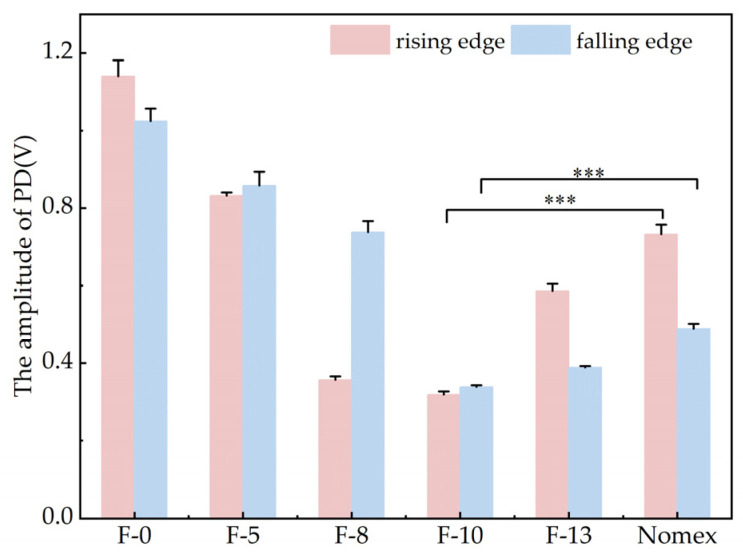
Variation curves of PD amplitude at the rising and falling edges for the column-plate mode (*** refers to p<0.001).

Based on the experimental setup described in Section 2.1, PD experiments were carried out using the step-up voltage method. The amplitudes of partial discharges for composite aramid paper in the needle-plate model and column-plate model at 5 kV (5 kHz) were obtained, as shown in Figure 7 and Figure 8, respectively. To facilitate the calculation of the average discharge amplitude for different filler contents, Figure 9 and Figure 10 are generated based on Figure 7 and Figure 8, respectively. As evident from the figures, the partial discharge amplitude initially decreases and then increases with increasing composite filler content. The PD amplitude for F-10 is the lowest, indicating that the composite aramid paper with a 10% filler content exhibits the greatest partial discharge resistance. Compared with Nomex paper, the average reduction in partial discharge amplitude was 59.65% for the needle-plate model and 25.48% for the column-plate model, respectively.

### 3.2. PD Duration

**Figure 11:**
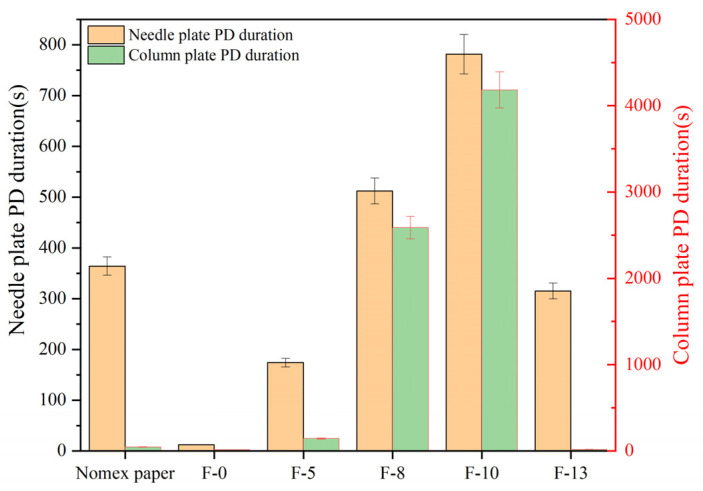
Duration of different materials at 20 kHz.

Based on the experimental setup described in Section 2.1, PD experiments were carried out using the constant voltage method. The partial discharge durations for composite aramid paper under 5 kV (20 kHz) in both the needle-plate and column-plate models are shown in Figure 11. As indicated in the figure, the partial discharge duration initially increases and then decreases with increasing composite filler content. The longest partial discharge duration is observed for F-10, which further confirms that the composite aramid paper with a 10% filler content exhibits the greatest partial discharge resistance. Compared with Nomex paper, the average partial discharge duration increased by 114.56% in the needle-plate model and 9195.56% in the column-plate model, respectively.

### 3.3. PD Breakdown Voltage

**Figure 12:**
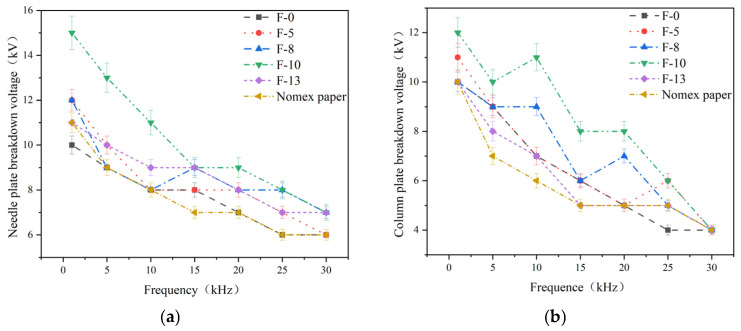
Breakdown voltage of different samples at high-frequency voltage: (**a**) Needle-plate model breakdown voltage; (**b**) Column-plate model breakdown voltage.

PD breakdown voltage refers to the applied test voltage on the composite aramid paper when it undergoes complete insulation breakdown. Based on the experimental setup described in Section 2.1, PD tests were conducted using the step-up voltage method. The breakdown voltages of composite aramid paper at different frequencies for both the needle-plate and column-plate models are shown in Figure 12. As indicated in the figure, for a given filler content, the breakdown voltage of composite aramid paper decreases with increasing applied frequency. At a given frequency, the breakdown voltage initially increases and then decreases with increasing nanofiller content, and F-10 exhibits the highest breakdown voltage. This further confirms that the composite aramid paper with a 10% filler content exhibits the greatest partial discharge resistance. Compared with Nomex paper, the average breakdown voltages in needle-plate and column-plate models increased by 32.2% and 38.5%, respectively.

**Figure 13:**
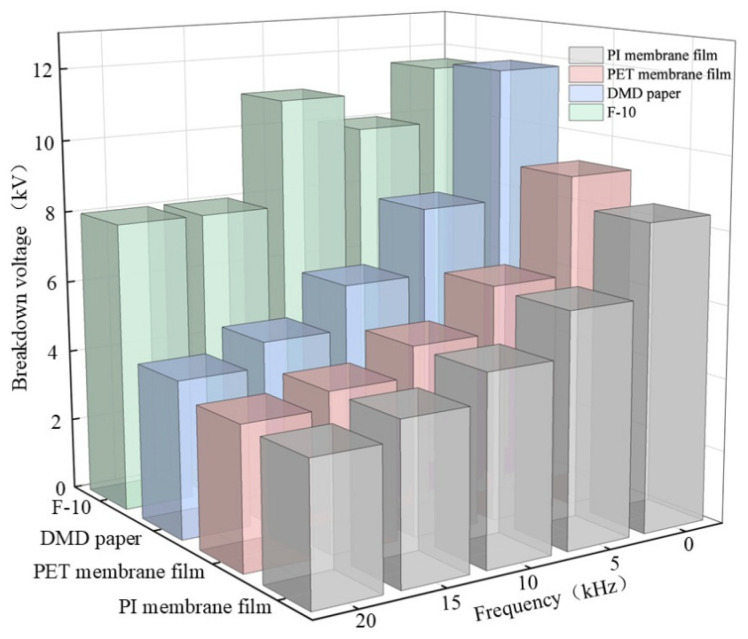
PD breakdown voltage of different materials.

Compared with other composite materials reported in the literature, as shown in Table 5 and Figure 13, the PD breakdown voltage of the F-10 sample prepared in this study is significantly higher. This improvement may be attributed to the effects of residual charges on the surface of composite aramid paper and the enhanced interfacial bonding between the matrix and the filler. On the one hand, the combined effects of the initial charge accumulation and the surface charge decay rate in composite aramid paper result in variations in the residual charges deposited on the surface. This, in turn, alters the electric field strength and carrier mobility at insulation defects, thereby modifying the PD characteristics of composite aramid paper. On the other hand, the enhanced interfacial bonding between the matrix and the fillers reduces internal defects and traps within the material. Under increasing electric field strength, electrons are less likely to escape from traps, thereby increasing the breakdown voltage and partial discharge duration while reducing the partial discharge amplitude.

## 4. Influence Mechanism

### 4.1. The Effect of Residual Charge

**Figure 14:**
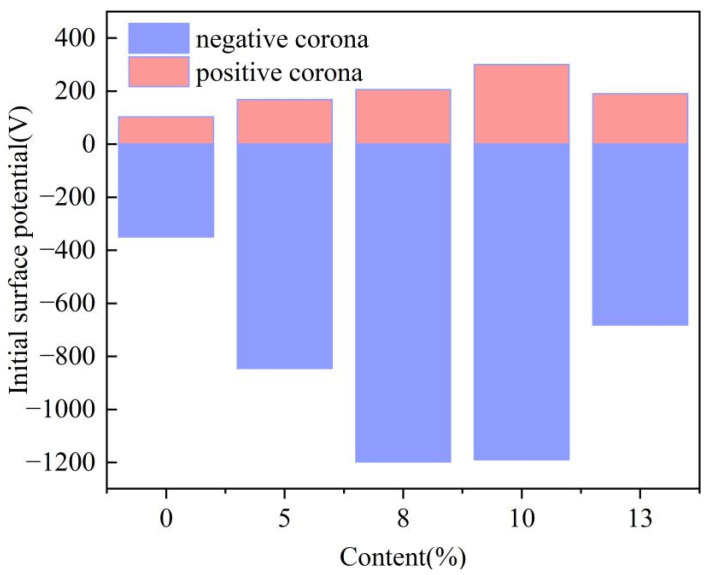
Distribution of initial surface potential for composite aramid paper with different filler contents.

To investigate the influence of residual charges on PD in composite aramid paper, surface potential tests were conducted using the surface potential measurement platform for composite aramid paper, as described in Section 2.2. The results for the initial surface potential and surface potential decay rate of composite aramid paper specimens are presented in Figure 14. As demonstrated in Figure 14, the initial surface potential of composite aramid paper specimens initially increases and then decreases with increasing filler content. Furthermore, the maximum initial surface potential is observed for a 10% filler content.

**Figure 15:**
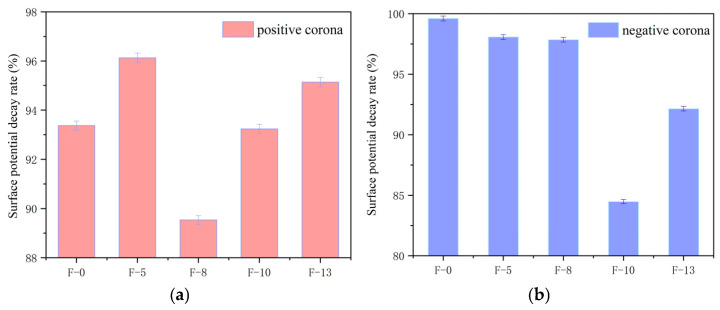
Surface potential decay rate of composite aramid paper with different filler contents: (**a**) Surface potential decay rate (%) under positive corona; (**b**) Surface potential decay rate (%) under negative corona.

In order to facilitate quantitative comparative analysis, the degree of potential decay is expressed using the surface potential decay rate *D* [29,30,31].

(2) $$D=\frac{V_{0}-V_{3000}}{V_{0}}\times 100\%$$

where *V*_0_ is the surface potential at the initial time and *V*_3000_ is the surface potential at a decay time of 3000 s. The obtained surface potential decay rate is shown in Figure 15.

As shown in Figure 15, the surface potential decay rate initially decreases and then increases with increasing filler content. After positive corona charging, the composite aramid paper with an 8% filler content exhibits the lowest surface potential decay rate (approximately 89.53%), whereas the composite aramid paper with a 5% filler content shows the highest decay rate (approximately 96.13%). After negative corona charging, the composite aramid paper exhibits the lowest surface potential decay rate (approximately 84.48%) at a filler content of 10%, whereas the highest decay rate (approximately 99.6%) occurs at a zero filler content.

**Figure 16:**
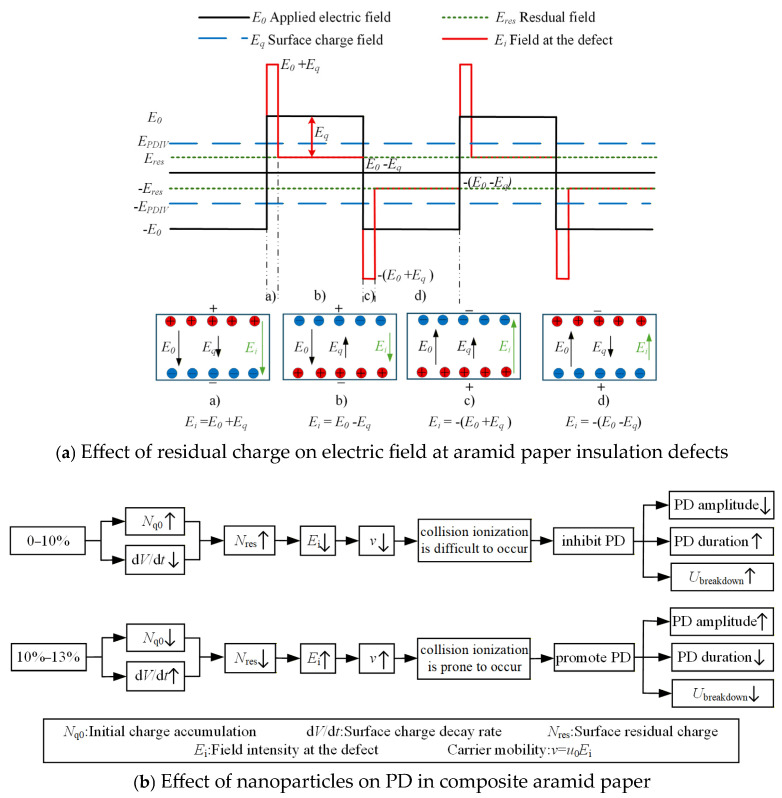
Effect of residual charge on PD in composite aramid paper.

Based on the aforementioned research findings, Figure 16 illustrates the mechanism by which residual charges influence PD behavior in composite aramid paper. As the composite particle content increases from 0% to 10%, the initial charge accumulation on the surface of composite aramid paper increases, whereas the surface charge decay rate decreases. The combined effects of these factors result in an increase in the residual charges deposited on the surface of composite aramid paper, along with reductions in the electric field strength at insulation defects and a decrease in carrier mobility. Consequently, collision ionization is less likely to occur, thereby suppressing the occurrence of partial discharges. This results in an elevated partial discharge breakdown voltage, an extended partial discharge duration, and a reduced partial discharge amplitude, as shown in Figure 7, Figure 8, Figure 9, Figure 10, Figure 11 and Figure 12.

As the filler particle content increases beyond 10% to 13%, the initial charge accumulation on the surface of composite aramid paper decreases, whereas the surface charge decay rate increases. The combined effects of these factors result in a reduction in the residual charges deposited on the surface of composite aramid paper, along with an enhancement of the electric field strength at insulation defects and an increase in carrier mobility. Consequently, collision ionization is more likely to occur, thereby promoting the occurrence of partial discharges. This leads to a decreased partial discharge breakdown voltage, a reduced partial discharge duration, and an increased partial discharge amplitude, as shown in Figure 7, Figure 8, Figure 9, Figure 10, Figure 11 and Figure 12.

### 4.2. The Effects of Hydrogen Bonds

**Figure 17:**
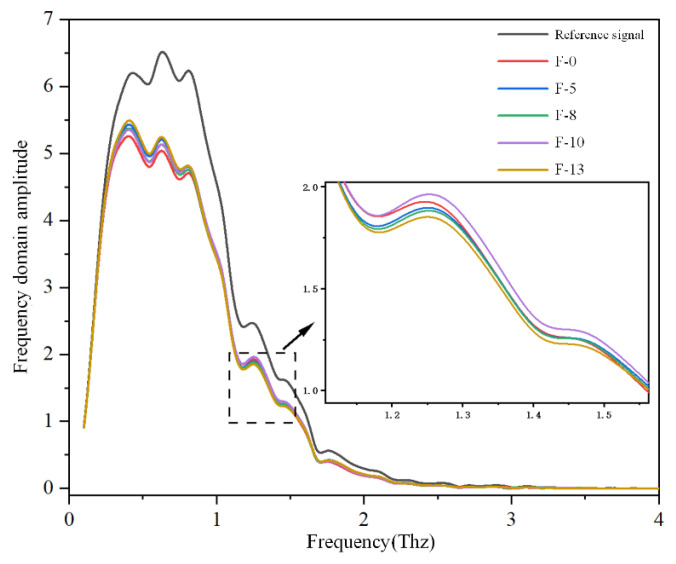
Terahertz-frequency-domain spectra of different samples.

In the terahertz frequency range, peak absorption primarily originates from weak interactions, such as hydrogen bonds or lattice vibrations. The formation or strengthening of hydrogen bonds increases the intensity of absorption peaks, whereas the breaking or weakening decreases it. Based on this principle, terahertz spectra of composite aramid paper with varying nanofiller contents were obtained using the terahertz detection platform described in Section 2.2.2, as shown in Figure 17.

**Figure 18:**
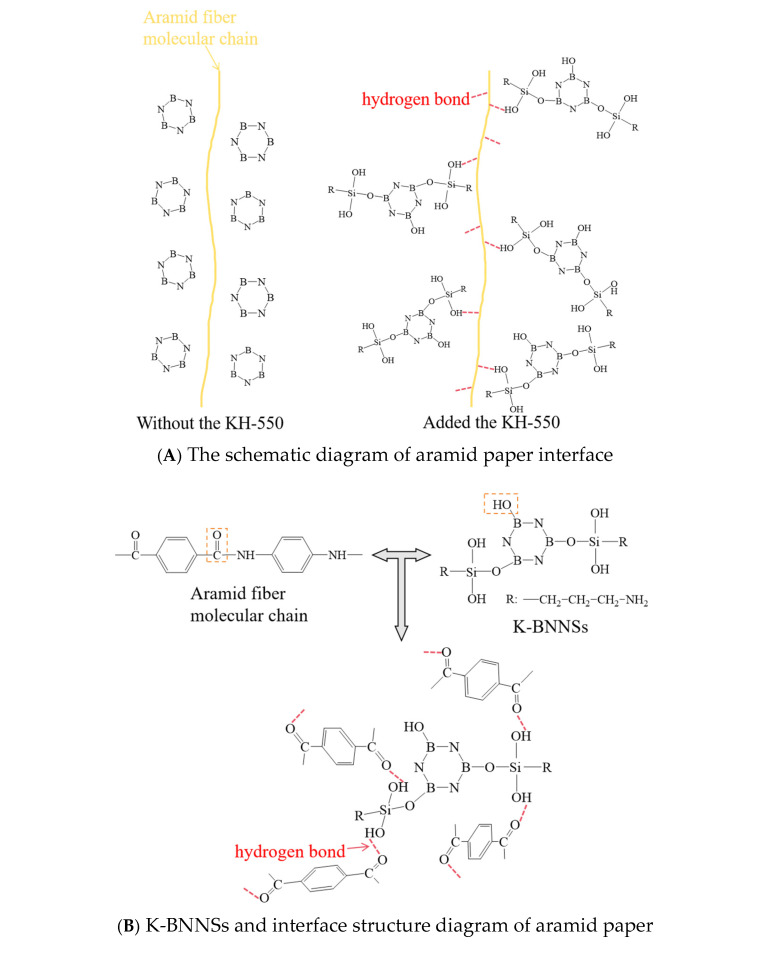

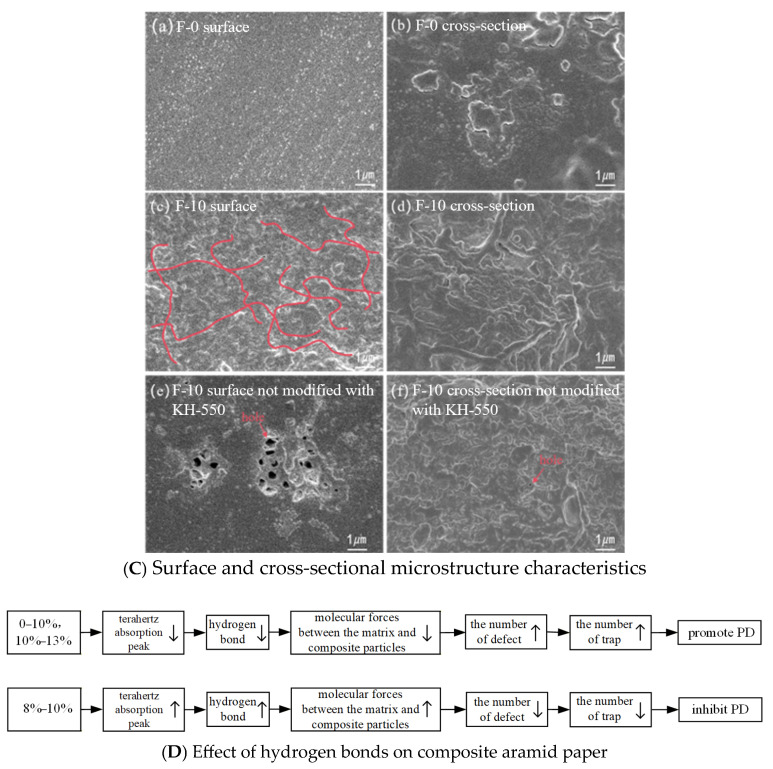
Mechanism of hydrogen bonds’ influence on PD in composite aramid paper.

As shown in Figure 17, all samples of composite aramid paper with different nanofiller contents exhibit a characteristic absorption peak at approximately 1.25 THz. This peak corresponds to the specific resonant absorption between interfacial hydrogen bonds and terahertz waves, as the 1–2 THz range lies in the typical characteristic region for the vibration of strong intermolecular/interfacial hydrogen bonds [32]. The positions of these characteristic absorption peaks are nearly identical across all five sample groups, indicating that the factors influencing terahertz wave absorption in composite aramid paper are consistent. As the filler content increases, the intensity of the characteristic absorption peak initially decreases, then increases, and subsequently decreases again. The F-10 sample, with a 10% filler content, shows the highest amplitude in the frequency domain, suggesting that, at a 10% filler content, weak interactions, such as hydrogen bonds, are the strongest or that the greatest number of hydrogen bonds formed is the highest. When molecular chains within the composite aramid paper vibrate or twist under a high-frequency square-wave electric field, the addition of KH-550 for a 10% filler content promotes the formation of hydrogen bonds between the composite particles and the aramid matrix. This hydrogen bonding effect is the strongest, thereby suppressing the occurrence of partial discharges. This results in an increased high-frequency partial discharge breakdown voltage and an extended partial discharge duration, while reducing the partial discharge amplitude. Consequently, the suppression of partial discharges is most pronounced, and the corresponding mechanism is illustrated in Figure 18. Furthermore, the conjecture in Reference [18] is experimentally validated. The marginal differences among samples are derived from gradient changes in hydrogen bonds driven by filler dispersibility, reproducible via parallel tests, cross-validated by SEM, and reflective of terahertz spectroscopy’s high sensitivity to weak intermolecular interactions.

As shown in Figure 18, for the F-10 specimen, KH-550 modification enables uniform nanofiller dispersion and the formation of optimal interfacial hydrogen bonds, which alleviates agglomeration of aramid pulp and yields an interlaced dendritic-layered structure. This results in a homogeneous matrix with tight interparticle bonding, thereby fostering the construction of an efficient thermal conductive network. F-8 suffers from insufficient interfacial hydrogen bonds due to poor nanofiller dispersion, while F-13 presents a disrupted hydrogen bond structure caused by severe nanofiller agglomeration. For the 10% unmodified K-BNNS specimen, the absence of modification-induced interfacial hydrogen bonds leads to poor filler-matrix compatibility, accompanied by pronounced voids and severe agglomeration, thus resulting in impaired construction of the thermal conductive network. The aforementioned interfacial hydrogen bond and microstructural characteristics are in excellent agreement with the terahertz absorption results of the specimens at 1.25 THz, with F-10 exhibiting the highest absorption peak intensity across all samples.

## 5. Conclusions

Based on the foregoing research and analysis of the influence of K-BNNS nanoparticles on the PD characteristics of composite aramid paper, the following key conclusions can be drawn:

(1) K-BNNS nanoparticles significantly improve the partial discharge performance of composite aramid paper. When the filler content reaches a 10% level, the composite aramid paper exhibits optimal insulation properties, with the lowest partial discharge amplitude, the longest partial discharge duration, and the highest partial discharge breakdown voltage.
(2) At a filler content of 10%, the composite aramid paper exhibits the highest residual charges on its surface, as well as the lowest electric field strength at defects and the lowest carrier mobility. This configuration inhibits collision ionization, thereby suppressing the occurrence of partial discharges.
(3) The introduction of KH-550 enhances the interfacial bonding strength between the composite particles and the aramid matrix. At a filler content of 10%, the hydrogen bonding forces are the strongest, internal material defects are minimized, and the suppression of partial discharges is most pronounced.

These results may guide the design and selection of insulation materials for high-frequency transformers, while also providing a theoretical basis and technical support for assessing the insulation state of high-frequency transformers.

## Figures and Tables

**Table 1:** Test platform parameters.

Equipment Name	Equipment Specifications	Manufacturer	Equipment Model
High-frequency pulse power supply	Voltage: Bipolar 0–20 kV; Frequency: 0–30 kHz;	Dalian Haifu Technology Co., Ltd.	P40
High-voltage probe	Voltage range: 0–40 kV; Bandwidth: 75 MHz; Attenuation ratio: 1000:1	Tektronix	P6015A
High-frequency current sensor	Operating frequency range: 10–500 MHz	WINTECH	HFCT-01
Oscilloscope	Bandwidth: 1 GHz; Sampling rate: up to 5 GS/s	Tektronix	MDO34-350

**Table 2:** Experimental conditions of high-frequecy PD test.

Applied Voltage (kV)	Step-Up Voltage (kV)	The Test Frequency (kHz)	The Rise Time (ns)	The Number of Repetitions
≤1	0.2	1, 5, 10, 15, 20, 25, 30	300	5
>1	1	1, 5, 10, 15, 20, 25, 30	300	

**Table 3:** Equipment parameters.

Equipment Name	Equipment Description	Manufacturer	Equipment Model
HF power	Voltage: Bipolar 0–20 kV; Frequency: 0–30 kHz Duty cycle: 0.02–100%	Dalian Hai Vo Technology Co.	P40
Data acquisition system	Accuracy: 0.0035%	GWINSTEK	DAQ-9600
Surface voltage measurements	Measurement range: 0–±20 kV	Trek	Trek-341B
Probe	Measurement accuracy: ±0.1%	Trek	3460-1

**Table 4:** Experimental conditions of surface potential test.

Applied Voltage (kV)	Test Frequency (kHz)	Repetition
5	1, 5, 10, 15, 20, 25, 30	5

**Table 5:** Insulation material parameter index.

Insulating Material	Nominal Thickness/mm	PD Duration/s
PI/Al_2_O_3_ [27]	0.015	351,000
PI/GAPD [15]	0.028 ± 0.002	274,752
EP/P-BN [16]	1.5 ± 0.2	42.05
BN/SiO_2_/Al_2_O_3_ [17]	0.5	42.21
BNNS-PDA/PI [28]	0.5	95,040
This article	0.05	70~80

## Data Availability

The data presented in this study are available on request from the corresponding author.
